# Reporting quality of Cochrane systematic reviews with Chinese herbal medicines

**DOI:** 10.1186/s13643-019-1218-y

**Published:** 2019-12-03

**Authors:** Xuan Zhang, Qi-Ying Aixinjueluo, Si-Yao Li, Lisa-L Song, Chung-Tai Lau, Ran Tan, Zhao-Xiang Bian

**Affiliations:** 1 Chinese Clinical Trial Registry (Hong Kong), Hong Kong Chinese Medicine Clinical Study Centre, School of Chinese Medicine, Hong Kong Baptist University, Kowloon Tong, Hong Kong; 2 Dr. Stephen Riady Chinese Medicine Library, Hong Kong Baptist University, Kowloon Tong, Hong Kong

**Keywords:** Cochrane systematic review (CSR), Chinese herbal medicine (CHM), PRISMA statement, Reporting quality, Traditional Chinese medicine (TCM), TCM principles, methods, formulas, and herbs

## Abstract

**Background:**

Chinese herbal medicines (CHMs) are the major interventions of traditional Chinese medicine (TCM), which are typically administered as either single herbs or formulas. The Cochrane systematic reviews (SRs) of CHMs are essential references for evaluating the efficacy and safety of CHMs interventions; they are expected to be accurate and reliable. This study aimed to assess the reporting quality of these SRs, particularly whether necessary information related to CHM was adequately reported.

**Methods:**

The Cochrane Database was systematically searched for all SRs of CHM that were published up to 31 December 2017. The primary analysis was to assess their reporting quality based on 27-item of the Preferred Reporting Items for Systematic reviews and Meta-Analyses (PRISMA) and 9-item of CHM-related information designed according to TCM theory. Descriptive statistics were additionally used to analyze their baseline characteristics.

**Results:**

A total of 109 Cochrane SRs of CHM were identified from 1999 to 2017. For 27-item of PRISMA, 26 had the reporting compliances higher than 50%, of which 11 were fully reporting (100%). However, for CHM-related information, 65 (59.6%) SRs did not report the specific name of the CHM in the title, 42 (38.5%) lacked TCM-related rationales in the introduction, 62 (56.9%) did not include CHM-related characteristics in the additional analyses, and 77 (70.6%) did not analyze CHM results in terms of TCM-related theories in the discussion. Of 97 SRs that included clinical trials, 38 (39.2%) did not provide the details of composition and dosage of CHMs, 85 (87.6%) did not report the CHM sources, 13 (13.4%) did not provide the dosage form, 95 (97.9%) lacked CHM quality control information, and 57 (58.8%) did not describe details of the controls. For 62 (72.9%) of 85 SRs that included meta-analysis, it was impossible to assess whether meta-analysis had been properly conducted due to inadequate reporting of CHM interventions.

**Conclusion:**

Although the Cochrane SRs of CHM showed reporting compliance with PRISMA checklist, their reporting quality needs improvement, especially about full reporting of CHM interventions and of TCM-related rationales. Reporting guideline of “PRISMA extension for CHM interventions” should be developed thus to improve their quality.

## Background

Systematic reviews (SRs) summarize large bodies of evidence and synthesize all relevant studies that address a specific clinical question [1]. A meta-analysis (MA) is a tool that uses statistical methods to quantitatively combine and summarize the results of several independent studies in an SR [2]. SR/MA can help clinicians keep up to date with their field and policymakers judge the risks and benefits of health care behaviors; they provide a starting point for clinical practice guideline developers and summaries for funders seeking new research to support [3]. As with individual research reports, the value of an SR/MA largely depends on its transparent reporting [4].

Unfortunately, there is considerable evidence that the reporting quality of SR/MA is often poorly, thus diminishing their potential usefulness [5–7]. The suboptimal reporting quality of SR/MA led to the development of the QUOROM (Quality Of Reporting Of Meta analyses) Statement and its updated revision named PRISMA (Preferred Reporting Items for Systematic reviews and Meta-Analyses), published in 1999 and 2009, respectively [8, 9]. The PRISMA Statement consists of a 27-item checklist and a four-phase flow diagram, with an explanation and elaboration for each checklist item also published in 2009 [10]. The PRISMA checklists are used to guide authors of SR/MA to improve reporting quality. It is also a universal criterion to assess the reporting quality of available SR/MA publications [11–13]. For example, Tian JH et al. used the PRISMA checklist to evaluate the reporting quality of SR/MAs published in 2014, including 100 from China and 100 from the USA, and found that the PRISMA score was 21.2 (China) and 20.6 (USA), respectively. The authors concluded that the quality of SR/MAs from both countries needs to be further improved [14].

The Cochrane Collaboration is an international organization that aims to prepare and maintain rigorous systematic reviews in order to help people make well-informed decisions about health care [15]. Some scholars have found that the Cochrane reviews appear to have greater methodological rigor, are more frequently updated, and are less prone to bias than other reviews published in non-Cochrane journals [16, 17]. Handoll H et al. have indicated that most Cochrane reviews are of a good standard [18]. For example, Fleming PS et al. used the AMSTAR (A MeaSurement Tool to Assess systematic Reviews) checklist to assess and compare the methodological quality of Cochrane SRs (e.g., published in Cochrane Database from January 2000 to July 2011) and non-Cochrane SRs (e.g., published in five leading orthodontic journals from February 2002 to July 2011). They finally identified 109 SRs, including 26 Cochrane SRs, and found that the Cochrane SRs showed higher levels of methodology quality than non-Cochrane SRs (*P* < 0.01) [19].

Chinese herbal medicine (CHM), an essential part of TCM and the typical representative of TCM interventions, is recognized, more and more, as having profound value because of its demonstrated curative effects [20]. CHM interventions include Chinese medicinal substances (single herbs) and CHM formulas (“*Fu*-*Fang*,” or specific combinations of generally more than two Chinese medicinal substances). Chinese medicinal substances mainly originate from natural sources, including plants, animals, minerals, and some chemical or biological products; they can be raw (fresh or dried) or processed, while the CHM formulas are combinations of Chinese medicinal substances that are either individualized or fixed, often traditional, now sometimes patented [21]. Since the first SR of CHM was published in 1997 [22], the number of SR/MA of CHM increased rapidly [23]. For example, Chen M et al. analyzed 218 SR/MA of CHM published in Chinese journals from 1998 to 2008 and found that 82.1% were CHM formulas (including both patent proprietary CHM formulas and individualized CHM formulas), 10.1% were Chinese medicinal substance (including single herbs and herbal extracts), and 7.8% were unspecified [24].

In terms of the reporting quality of SR/MA of CHM, many studies have examined the compliance with PRISMA 27 items and have concluded similarly that the quality of SR/MA published in Chinese journals is poor and needs much improvement [25–27]. Also, the poor-quality reviews have been criticized for addressing too broadly defined topics and selecting too many different kinds of herbal medicines and formulas, thus leading to bias in the results [28, 29]. Although the quality of data provided in the primary clinical trials affects the quality of results in the SR/MA, proper synthesis of data and sufficient analysis of potential bias are also essential to determine the overall quality of the final results of SR/MA [30]. Therefore, it is necessary to collate the original information related to CHM, considering their source, dosage, duration, processing method, composition, and form of the CHM intervention, in order to make sure whether the interventions used in different clinical trials are the same [31]. However, no previous study has assessed whether CHM-related information is sufficiently collected and reported in the published SR/MA of CHM; nor has any study identified what key information related to CHM is the basis of the synthesis of results, especially for meta-analysis [32, 33].

Given the importance of reporting CHM-related information in SR/MA, a first step would be a systematic survey of CHM SRs to identify the common problems, if any. As Cochrane reviews are usually noted to have better methodology quality, this study aimed to examine the reporting of Cochrane reviews of CHM. In this study, the assessments of reporting quality were not only based on the standard 27-item of PRISMA but also on the 9-item of CHM-related information which specially designed according to the TCM theories of principles, methods, formulas, and herbs (also called “*Li*-*Fa*-*Fang*-*Yao*”). In clinical practice, the CHM treatment determination is usually guided with TCM theories of principles, methods, formulas, and herbs [34]. With reference to the reporting guidelines of CHM interventional trials [35], such as “CONSORT Extension for CHM Formulas 2017” [36], we have developed the 9-item checklist which reflecting CHM-related information and TCM rationale.

Therefore, this study had the following objectives: (a) to summarize the general characteristics of all included Cochrane SRs of CHM, (b) to assess the reporting quality of these SRs based on the PRISMA checklist, (c) to evaluate whether necessary information related to CHM is adequately reported, and (d) to assess whether these SRs are properly conducted in terms of synthesis of results (e.g., meta-analysis).

## Methods

### Inclusion and exclusion criteria

This study included all SR/MAs of CHM published in the Cochrane Library until 31 December 2017. The CHM interventions are typically administered as either Chinese medicinal substances (namely single herbs) or CHM formulas (namely “*Fu*-*Fang*”). Various dosage forms of CHM interventions, such as decoction, granule, pill, tablet, capsule, powder, medicated tea, medicated wine, oral liquid, plaster, and injection, were included. CHM interventions may have been administered alone or in combination with other interventions of conventional Western medicine or complementary alternative medicine. There were no limitations in the participants, comparisons, and outcomes. We excluded the following SR/MAs: repeat publications, comprehensive interventions focused on pharmacological treatment rather than herbal medicine, non-herbal TCM interventions (e.g., acupuncture, moxibustion, Taichi), non-TCM herbal medicine (e.g., Tibetan, Japanese), plant extracts (e.g., plant-derived chemicals or synthetic chemicals which contain constituents of plants), protocols, and withdrawal SR/MAs.

### Search strategy

The Cochrane Database of Systematic Reviews was searched on 29 May 2018 for all Cochrane SR/MAs of CHM that had been published up to 31 December 2017. The search terms included “Chinese herbs,” “Chinese medicine,” “herb,” “traditional herbal medicine,” “Chinese materia medica,” “Chinese medicine prescription,” “formula,” “Chinese patent,” etc. The detailed search strategy is given in Additional file 1: S_1_.

### Screening

The titles and abstracts of the SRs were independently screened by two researchers (XZ and Q-YA) based on inclusion and exclusion criteria, and the full-texts of potentially suitable articles were retrieved for further assessment. Disagreements were resolved by a third reviewer (Z-XB).

### Data extraction

There were three pre-designed forms for data collection: (1) General characteristics form, including publication year, information of the authors, and descriptive information of included SRs. (2) PRISMA assessment form, including 27 items of the checklist. (3) CHM-related information form, which was designed according to (i) the reporting guidelines of “CONSORT Extension for CHM Formulas 2017”; and (ii) the TCM theories of principles, methods, formulas, and herbs. Aiming for easy calculation, the specifics of CHM-related information were categorized into nine items, including title, introduction, information source, eligibility criteria for participants and outcomes, study characteristics (for CHM interventions and control groups), additional analyses, synthesis of results, and discussion. The details are presented in Table 1.

**Table 1:** Nine items for reporting assessment on CHM-related information

Item 1: Whether a specific name of the CHM intervention(s) was reported in “Title” section? Item 2: Whether the CHM-related rationale was included in the “Introduction/Background” section? Item 3: Whether the Chinese database(s) and/or journals was included in the search strategy in “Information source”? Item 4: Whether the TCM diagnostic criteria (e.g., TCM pattern/syndrome) was included in the “Eligibility criteria for participants”? Item 5: Whether TCM-related outcomes (e.g., pattern scores) were included in the “Eligibility criteria for outcome measures”? Item 6: Whether the CHM intervention details, including composition and dosage, type, dosage form, source, administration route, time of administration, and quality control of the CHM were reported in the “Characteristics of included studies” section? For comparison, whether the details of controls were reported? Item 7: Whether the CHM characteristics were considered in the subgroup analysis, sensitivity analysis or other analysis of clinical heterogeneity in “Additional analyses” section? Item 8: Whether the heterogeneity of CHM formula, such as composition and dosage, has been fully considered when doing the data synthesis, especially about the meta-analysis? Item 9: Whether the relevant TCM theory was reported in the “Discussion” section?	

### Data analysis

The 27-item of PRISMA and the 9-item of CHM-related information were used as the tools for assessing reporting quality. Although some items included several subitems, the scoring criteria is consistent, namely each item or subitem was given a “1” score if fully reported or “0” if incompletely reported or absent. The specific methods for scoring each item/subitem are presented in Additional file 1: S_2_. In order to increase the accuracy of scoring, the predefined scoring rules were tested on 20 random SRs (approximately 20% of included SRs) first, and then subsequently used to assess all SRs. After the scoring rules were determined, two researchers (XZ and Q-YA) assessed the SRs independently, and the results were double-checked. Any problems or ambiguities were resolved by discussion with third review (Z-XB). All data were collected and recorded in Microsoft Office Excel (Version 2016). Categorical data is presented as a number (*n*) and percent (%).

## Results

### Search

**Fig. 1:**
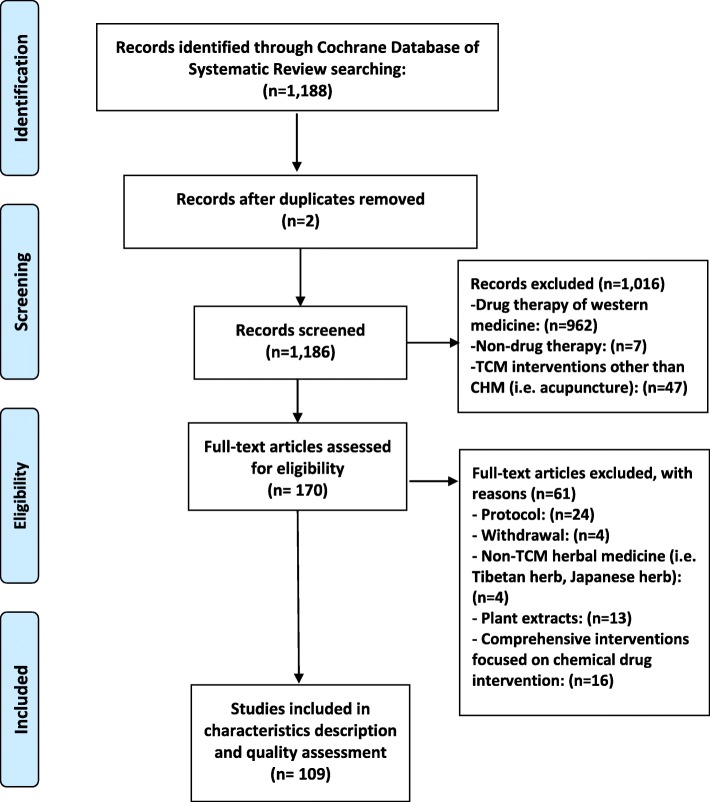
Flow chart of CDSR in CHM

Our initial literature search identified 1188 records. Preliminary screening excluded 1018 SRs due to duplication or focus on non-CHM interventions. After examination of the full texts of 170 articles, a total of 109 SRs was eligible for inclusion in this study (Fig. 1).

### General characteristics of included SRs

**Fig. 2:**
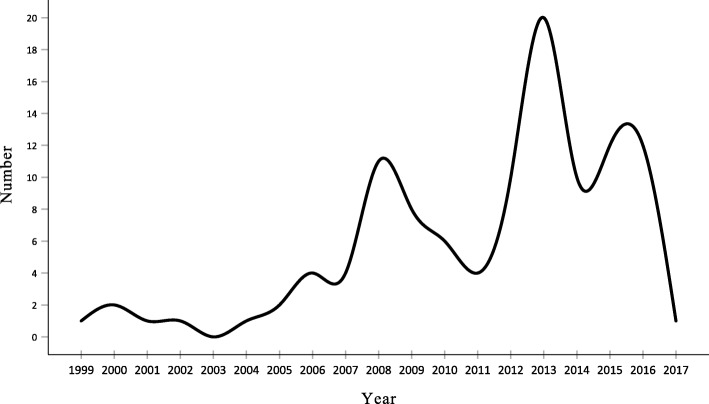
The number of included CDSR in CHM from 1999 to 2017

The earliest Cochrane SR of CHM was published in 1999. Since 2005, the number of SRs has increased markedly, especially in 2013. More than half (58.7%, 64/109) of the SRs were published between 2012 and 2016 (Fig. 2).

Table 2 presents the general characteristics of included SRs, involving the number of authors, background of the first author, number of included randomized controlled trials (RCTs) and participants, etc. Most of SRs (97.2%) published protocols, and 78.0% SRs conducted meta-analysis. Of 109 SRs, 45.0% have been periodical updated, and 43.1% has been cited more than five times.

**Table 2:** General characteristics of included SRs

Category	Descriptive characteristics	*N* (%)
Meta-analyses	Yes	85 (78.0)
Number of authors included	2–5	75 (68.8)
	6–10	31 (28.4)
	> 10	3 (2.8)
Background of the first author	Clinician	44 (40.4)
	Researcher/Methodologist	65 (59.6)
	Possess TCM background	27 (24.8)
Institution of the first author	Hospital	35 (32.1)
	University	74 (67.9)
	Institution with EBM center	67 (61.5)
Geographical distribution (corresponding author)	Mainland China	72 (66.1)
	Australia	15 (13.8)
	United Kingdom	11 (10.1)
	Hong Kong	6 (5.5)
	Others^a^	5 (4.6)
Types of primary studies included	RCTs	109 (100)
Number of included RCTs	0	12 (11.0)
	1–20	71 (65.1)
	21–50	21 (19.3)
	> 50	5 (4.6)
Number of included participants	0	12 (11.0)
	1–300	14 (12.8)
	301–500	13 (11.9)
	501–1000	23 21.1)
	1000–5000	36 (33.0)
	> 5000	10 (9.2)
	Unclear	1 (0.9)
Funding source	Yes	94 (86.2)
Number of times cited	0	14 (12.8)
	1–5	48 (44.0)
	6–10	29 (26.6)
	11–20	14 (12.8)
	> 20	4 (3.7)
Update of a previous review	Yes	49 (45.0)
	No (SRs published before 2012)^b^	28 (25.7)
Had protocols published	Yes	106 (97.2)

^a^Others including Canada (1), Germany (1), Netherlands (1), USA (2)

^b^There were 60 SRs had not been updated, and we calculated the number of these SRs which published before January 1, 2012

### Targeted diseases and conclusions of included SRs

As shown in Table 3, the three most frequently examined conditions were diseases of the circulatory system, genitourinary system, and certain infectious and parasitic diseases (14.7%, 13.8%, and 11.0%, respectively). Most (95.4%) SRs concerned the efficacy and safety of CHM interventions, of which 45.9% treatments included both single herbs and CHM formulas. For conclusions, only six SRs draw a certain conclusion (positive or negative); the remaining 103 SRs cannot do that, although 32 of them tended to conclude that CHM interventions were beneficial, the low quality of RCTs is the primary limitation.

**Table 3:** Descriptive information of included SRs

Category	Descriptive characteristics	*N* (%)
Types of CHM interventions	Chinese single herb(s)	32 (29.4)
	CHM formula(s)	25 (22.9)
	Both single herbs and CHM formulas	50 (45.9)
	Not specific^a^	2 (1.8)
Types of diseases^b^	Diseases of the circulatory system	16 (14.7)
	Diseases of the genitourinary system	15 (13.8)
	Certain infectious and parasitic diseases	12 (11.0)
	Endocrine, nutritional and metabolic diseases	10 (9.2)
	Diseases of the nervous system	9 (8.3)
	Pregnancy, childbirth and the puerperium	8 (7.3)
	Diseases of the respiratory system	7 (6.4)
	Diseases of the digestive system	6 (5.5)
	Mental and behavioral disorders	6 (5.5)
	Diseases of the musculoskeletal system and connective tissue	6 (5.5)
	Neoplasms	6 (5.5)
	Diseases of the skin and subcutaneous tissue	5 (4.6)
	Injury, poisoning and certain other consequences of external causes	2 (1.8)
	Diseases of the ear and mastoid process	1 (0.9)
Problem concerned^c^	Treatment	104 (95.4)
	Prevention	3 (2.8)
	Both treatment and prevention	2 (1.8)
Review authors’ conclusion^d^	Certainty of positive effect	3 (2.8)
	Uncertain but may be beneficial	32 (29.4)
	Unclear/ lack of evidence	71 (65.1)
	Certainty of negative effect	3 (2.8)

^a^The interventions were reported as Chinese medicine or traditional Chinese medicine, but did not specify either the single herb or formula used

^b^According to International Statistical Classification of Diseases and Related Health Problems 10th Revision (ICD-10) Version for 2010

^c^Problem concerned refers to the objectives of included SRs

^d^Certainty of positive effect—significant benefit found or at least one CHM intervention recommended; Uncertain but may be beneficial—the CHM interventions studied offer possible benefits but the current evidence is insufficient to draw definitive conclusions; unclear/lack of evidence—lack of reliable evidence to judge or evaluate; certainty of negative effect—no significant benefit found, or no CHM intervention recommended

### PRISMA checklist score

As presented in Table 4, except the title requirement (item 1) of PRISMA was not applicable for Cochrane SRs, the total reporting rates of the remaining 26 items varied from 54.1 to 100%. Eleven items, including item 2–4, 6, 13, 18–21, 24, and 26 were fully reported (100%). Thirteen items, namely item 5, 7–10, 12, 14–17, 22, 25, and 27 were reported in more than 70% of all SRs. In comparison, only item 11 and 23 were reported in almost 50% of all SRs.

**Table 4:** Reporting quality of 27 items of PRISMA (*n* = 109 SRs)

Category	Item	Score, *n* (%)
Title	1. Title	0 (0%)^a^
Abstract	2. Structured summary	109 (100)
Introduction	3. Rationale	109 (100)
	4. Objective	109 (100)
Methods RESULTS	5. Protocol and registration	106 (97.2)
	6. Eligibility criteria	109 (100)
	7. Information sources	106 (97.2)
	8. Search	85 (78.0)
	9. Study selection	108 (99.1)
	10. Data collection process	107 (98.2)
	11. Data items	60 (55.0)
	12. Risk of bias in individual studies	108 (99.1)
	13. Summary measures	109 (100)
	14. Synthesis of results	107 (98.2)
	15. Risk of bias across studies	83 (76.1)
	16. Additional analyses	94 (86.2)
	17. Study selection	107 (98.2)
	18. Study characteristics	109 (100)
	19. Risk of bias within studies	109 (100)
	20. Results of individual studies	109 (100)
	21. Synthesis of results	109 (100)
	22. Risk of bias across studies	79 (72.5)
	23. Additional analysis	59 (54.1)
Discussion	24. Summary of evidence	109 (100)
	25. Limitations	83 (76.1)
	26. Conclusions	109 (100)
Funding	27. Funding	94 (86.2)

^a^Not applicable. The format of titles in Cochrane SRs is not required to include the words of “systematic review” or “meta-analysis”

### Reporting quality of CHM-related information

As presented in Table 5, nine items of CHM-related information were assessed. Of 109 SRs, 40.4% reported the specific names of CHM in the title, such as “*Oral Astragalus* (*Huang qi*),” “*Danshen* (*Chinese medicinal herb*)” for single herbs, or “*Wendan decoction* (*Traditional Chinese medicine*),” “*Chinese herbal medicine suxiao jiuxin wan*” for CHM formulas. Further, 83.5% SRs had searched Chinese databases or journals, and the common databases were CNKI (National Knowledge Infrastructure), VIP (VIP Chinese Science and Technique Journals Database), CBM (Chinese Biomedical Database), and Wanfang Database.

**Table 5:** Reporting quality of 9 items of CHM-related information (*n* = 109 SRs)

Category	Item	Specifics	Yes, *n* (%)
Title	1. Title	Specific name of CHM intervention	44 (40.4)
		Generalized name of CHM intervention^a^	58 (53.2)
		The name of multiple interventions including CHM^b^	7 (6.4)
Introduction	2. Rationale	TCM-related theory^c^	67 (61.5)
Methods	3. Information source	Chinese database^d^	88 (80.7)
		Chinese medical journals (hand-search)	23 (21.1)
		Chinese pharmaceutical company publications (hand-search)	3 (2.8)
		No Chinese database or journals reported	18 (16.5)
	4. Eligibility criteria for participants	Included TCM pattern/syndrome diagnosis criteria	2 (1.8)
	5. Eligibility criteria for outcomes	Included TCM-related outcomes	4 (3.7)
	6.Additional analyses	Considered CHM-specific characteristics	47 (43.1)
Results	7. Study characteristics^e^ (*n* = 97)	For CHM interventions	
		Composition and dosage	59 (60.8)
		Type of CHM	93 (95.9)
		Dosage form	84 (86.6)
		Source of CHM	12 (12.4)
		Administration route	89 (91.8)
		Time of administration	95 (97.9)
		Quality control of CHM	2 (2.1)
		For control groups	
		Adequate reporting	40 (41.2)
	8. Synthesis of results^f^ (*n* = 85)	Meta-analyses were properly conducted^g^	23 (27.1)
Discussion	9. Summary of evidence and limitations	Included the TCM theories	32 (29.4)

^a^Such as “Chinese herbal medicines,” “herbal medicines,” “herbal preparations,” “medicinal herbs,” “traditional Chinese medicine herbs,” etc.

^b^Such as “Interventions,” “Complementary therapies,” etc. CHM interventions were included in the full-texts

^c^For Cochrane SRs, the “Introduction” refers to the “Background”

^d^Specific calculation: one database (13 SRs), two databases (13 SRs), three databases (14 SRs), four databases (17 SRs), five databases (16 SRs), six databases (8 SRs), seven databases (1 SR), eight databases (1 SR), ten databases (1 SR)

^e^Because 12 SRs included no RCTs (as presented in Table 2), the percentage of “study characteristics” were based on the total number of 97. Take the first subitem (Composition and dosage) for example, 60.8% = 59/97

^f^Of 109 included SRs, 85 had meta-analysis (as presented in Table 2). Thus, to calculate the proportion of this item, the percentage of records was based on the total number of 85. For example, 27.1% = 23/85

^g^The criteria of “properly conducted” was according to the homogeneity of the PICO (e.g. participant, intervention, comparison and outcome) information, especially the reporting quality of the details of CHM interventions and additional analyses provided as above. For example, if some of the CHM-related information was not reported (e.g., CHM composition, dosage, source or quality control information), it is impossible to assess whether the meta-analyses in the SRs were properly conducted or not

In CHM SRs, the TCM-related rationale, diagnostic criteria, and outcome(s) were inadequately reported in the Introduction (61.5%), Discussion (29.4%), Eligibility criteria for participants (1.8%), and for outcomes (3.7%). The details of CHM interventions were also reported insufficiently, especially in terms of composition and dosage (60.8%), source (12.4%), and quality control of the CHM (2.1%). Besides, less than half (43.1%) SRs conducted additional analysis (e.g., subgroup analysis) based on specific CHM characteristics. Thus, for 85 SRs with meta-analysis, it is impossible to evaluate whether the synthesis of results had been appropriately conducted in 62 SRs (72.9%) due to their inadequate reporting in CHM treatments.

## Discussion

### General characteristics of included Cochrane SRs

In this study, we included 109 Cochrane SRs of CHM from 1999 to 2017 and described the baseline characteristics. Some problems have been discovered. Firstly, few authors of SRs have TCM relevant background. Most first-authors were clinicians and methodologists; however, only 25% (27/109) of them had TCM-related experience. For a person doing an SR of CHM, having TCM knowledge is better because they might well identify the clinical heterogeneity of different kinds of CHM interventions. Some scholars have indicated that it could be better to include professionals with TCM-related background in the author group of an SR of CHM [37]. Secondly, less than half of SRs have updated on time. Although it is well known that results from SRs are most useful when they are current, this study found that 55% (60/109) had not been timely updated, especially 28 SRs were published more than 5 years ago (namely before 2012). Moher D et al. have pointed out that an updating usually occurs after a certain period, such as 5 years, has passed since the completion of the original (or already updated) systematic review [38]. Generally, Cochrane SRs have a better record of updating than other SRs published in Chinese journals or other international journals; however, as indicated in this study, it still has room for improvement. Thirdly, nearly half (45.9%) of SRs selected a broad category of CHM single herbs and formulas. Indeed, choosing broad types of CHM interventions for one disease in an SR requires more rigorous methodology techniques in data analysis and combining to ensure reasonable results synthesis. Unfortunately, some scholars have found that as the proportion of broad selection of CHM interventions rises, and the rates of unreasonable synthesis of results more easily appeared [39].

### PRISMA score of included Cochrane SRs

For 27 items of the PRISMA, 18 items were well reported (> 90%). Except for the item 1 (title) was not applicable for Cochrane SRs, only two items, namely item 11 (Data items) and 23 (additional analyses), were reported relatively less frequently (nearly 55%). For SRs, whether original data items were extracted entirely and accurately is closely related to the synthesis of results. According to the characteristics of data items, whether the additional analyses (e.g., subgroup analysis) were properly designed is the key for assessing the value of summary results. For CHM SRs, the specific reporting items of CHM interventions are not provided in the standard PRISMA checklist, so there might be a gap between the international reporting guideline and specific reporting of CHM SRs. Thus, we have further assessed the reporting of CHM-related information based on a self-designed checklist.

### CHM-related information assessment of included Cochrane SRs

This study is the first attempt to assess the reporting quality of CHM-related information based on a self-designed checklist. As a result, we found that the inadequate reporting of CHM interventions and TCM-related rationales are needed to be improved urgently.

Firstly, for the reporting of CHM interventions, including type, dosage form, administration route and time, composition and dosage, source, and quality control, only nine SRs (8.3%) had a 100% reporting rate of these items. The least frequently reported information was the quality control (2.1%), and the CHM source (12.4%). We understand that such details may be not reported in the primary studies (e.g., RCT) [40], but they are necessary for judging consistency and heterogeneity of CHM interventions in the SRs [41]. If these details had not been collated completely, the combination of data must be interpreted with caution [42].

Secondly, the TCM rationales were inadequately reported in the included SRs. In clinical practice, the determination of CHM treatments should be guided by TCM theory of pattern differentiation [34]. Pattern (also called syndrome) differentiation is a critical component of TCM diagnosis and treatment; it is the main feature distinguishing it from Western medicine. Further, pattern differentiation refers to analyzing and summarizing the clinical symptoms gained by the four diagnostic methods of TCM (inspection, auscultation and smell, inquiry, and pulse taking and palpation) [43]. Similar to our results, previous studies have indicated that the CHM SRs published in Chinese journals had a poor reporting of TCM theories in the part of results discussion [44].

Thirdly, TCM-related diagnostic criteria and outcomes were rarely adopted in the included SRs. In CHM interventional clinical trials, it is common to adopt TCM pattern diagnostic criteria and relevant outcomes together with the Western medicine indicators [45]. The information of TCM pattern(s) is, however, often omitted in the final reports of SRs. If an SR of CHM does not consider TCM pattern diagnosis criteria or utilize TCM-related outcome(s), the summary results on the efficacy of CHM interventions may not be appropriately analyzed [46]. Authors of an SR should report the diagnostic criteria and/or outcomes of TCM-related factors, especially when these factors were used in the included RCTs of the SR.

Finally, due to the inadequate reporting of CHM interventions, more than half (57%, 62/109) SRs did not conduct subgroup analysis based on different features of CHM interventions (e.g., type, form, dosage). Moreover, for 85 SRs with meta-analysis, 73% (62/85) was impossible to assess whether data synthesis had been conducted properly. The synthesis of results includes statistical, methodological, and clinical considerations. Although the former two factors are perhaps more technical and evidence-based, the clinical considerations should be highly valued, especially for CHM treatments. According to different characteristics or categories of the CHM interventions (if any), the proper solution might be set subgroup analysis. For meta-analysis, it should be conducted under the condition of no heterogeneity between CHMs used in different trials [47].

### Improvement measures and suggestions

As some deficiencies of reporting were identified in this study, specific improvements are needed, because inadequate reporting of CHM interventions in the primary RCTs can directly compromise data collection and reporting quality of SRs [48]. Firstly, before starting an SR of CHM, it is necessary for the researchers to (a) consider the CHM-related rationale based on the TCM principles, methods, formulas, and herbs; and (b) design the form to extract the data about the study characteristics with sufficient CHM-related information (e.g., CHM intervention details).

Secondly, during the conduction of the SR, the authors need to (a) examine the heterogeneity of the participants, interventions, comparisons, outcomes (PICO) under the consideration of CHM characteristics; and (b) extract adequate information from the included clinical trials or contact their authors for inadequate or missing details. If the information cannot be obtained by contacting authors, then authors of an SR should describe this information as “not reported” [49]. We understand the data from original clinical trials with CHM should be improved too. For the reporting of RCTs, our working group has published the “CONSORT Extension for CHM formula: recommendations, explanation, and elaboration” in 2017, which can help authors standardize and improve the reporting quality of RCTs with CHM formula interventions [36].

Thirdly, the clear requirements about the CHM interventions and relevant rationales can improve the reporting quality of SRs with CHM. Existing PRISMA checklist did provide the requirements about the intervention. Because of the specificity of CHM, the readers may not provide the sufficient information about CHM. The CHM-specific items, such as TCM rationale, pattern (if any), outcomes, and details of CHM interventions, cannot be adequately captured by those items designed or written for typical healthcare SRs [50]. In other words, although achieving the full completeness of the PRISMA checklist, the current reporting quality of CHM SRs is still not optimal. Therefore, a reporting guideline of SRs for CHM interventions is necessary to be developed as an extension of the PRISMA checklist. This may improve the reporting quality of SRs of CHM. The guideline of “PRISMA extension for CHM interventions” should include a series of reporting items related to CHM interventions, and should reflect the characteristics of TCM principles, methods, formulas, and herbs. Our group has initiated the related work, and pre-registered this reporting guideline on the EQUATOR (Enhancing the QUAlity and Transparency Of health Research) in August 2016 [51]. We wish to finish the development soon.

## Limitations

This study has some limitations. Firstly, the results of this study were limited to Cochrane SRs and therefore may not be applicable to SRs published in other journals. Because Cochrane SRs are generally of better quality, problems in other SR publications may be even worse. Secondly, this study assessed the reporting quality of SRs of CHM mainly according to the PRISMA checklist and self-designed items of CHM-related information, and the assessment scoring (“1” or “0”) did not allow partial information to be used. All incomplete reporting (e.g., partial and absence) were given as “0”. This might influence the results due to artificial factors.

## Conclusion

CHM interventions, as either single herbs or formulas, are the primary type of TCM treatments. SRs of CHM summarize evidence relating to efficacy and safety of CHM interventions—but they are valuable only if done accurately and reliably. Although Cochrane SRs of CHM had compliance with the PRISMA checklist, their reporting quality still needs improvement in full reporting of CHM intervention details and related TCM rationales. This could be achieved by extending the PRISMA checklist to include CHM specific which are based on the unique characteristic of TCM principles, methods, formulas and herbs.

## Supplementary information

**Additional file 1.** S_1_ Search strategy. S_2_ Quality assessment rules of included CDSRs.

## Data Availability

The data used for this study is available from the corresponding author upon receiving a reasonable request.

## Abbreviations

CHM: Chinese herbal medicine;
CONSORT: Consolidated Standards of Reporting Trials
CSR: Cochrane systematic review
EQUAOR: Enhancing the QUAlity and Transparency Of health Research
MA: Meta-analysis
PICOS: Participant, Intervention, Control, Outcome, and Study design
PRISMA: Preferred Reporting Items for Systematic reviews and Meta-Analyses
QUOROM: Quality Of Reporting Of Meta analyses
RCT: Randomized controlled trial
SR: Systematic review
TCM: Traditional Chinese medicine
