# Supplementary material for: Reporting quality of Cochrane systematic reviews with Chinese herbal medicines
Source: Syst Rev. 2019 Dec 3;8:302. doi: 10.1186/s13643-019-1218-y (PMC6892158; doi:10.1186/s13643-019-1218-y)
Supplement: Supplementary file 1 — Additional file 1. S1 Search strategy. S2 Quality assessment rules of included CDSRs. [file 13643_2019_1218_MOESM1_ESM.pdf]

S_1: Search Strategy.

----

1 (chinese medicine or herb$).mp.

2 [exp Medicine, Traditional/]

3 ethnobotany.mp. or exp Ethnobotany/

4 phytotherapy.mp. or exp Phytotherapy/

5 Plant Extracts.mp. or exp Plant Extracts/

6 medicinal plant.mp. or exp Plants, Medicinal/

7 chinese herbal drugs.mp. or exp Drugs, Chinese Herbal/

8 chinese traditional medicine.mp. or exp Medicine, Chinese Traditional/

9 herbal medicine.mp. or exp Herbal Medicine/

10 (medicinal herb or pharmaceutical plant).mp.

11 (Chinese medicine$ or traditional medicine$).mp.

12 (herbal drug$ or herbal medicine$).mp.

13 (medicinal plant$ or medicinal herb$).mp.

14 (herb$ or herb$ formula$ or decoction$).mp.

15 (herb$ granule$ or herb$ capsule$ or herb$ pellet$).mp.

16 materia medica.mp. or exp Materia Medica/

17 or/1-16

18 ((single#entity or single) adj3 (component or drug$ or herb$)).mp.

19 (compound prescription$ or herbal mixture or Fufang).mp.

20 (Chinese Medicine Patent Prescription or proprietary Chinese medicines).mp.

21 (Chinese patent adj3 (medicine or drug$)).mp.

22 (Chinese adj3 (patent or proprietary) adj3 (medicine or drug$)).mp.

23 (Chinese adj2 (patent or proprietary) adj2 (medicine or drug$ or prescription$)).mp.

24 or/18-23

25 17 or 24

S2: Reporting quality assessment rules of included CDSRs.

Table 1. Assessment rules for the PRISMA checklist

Category	Item		Definition of "yes"
Title	1	Title	It should contain a word as "systematic review", "meta-analysis", or both. But in this study, item 1 was not assessed in the CDSRs.
Abstract	2	Structured summary	There was a structured summary including, as applicable, background, objectives, method (data sources, study eligibility criteria, participants, interventions, study appraisal and synthesis methods), results, conclusions and implications of key findings.
Introduction	3	Rationale	Described the rationale for the review in the context of what is already known. For the CDSRs, this content could be found in the section of background including "Description of the condition and intervention; How the intervention might work; and Why it is important to do this review".
	4	Objective	Provided the information of questions being addressed with reference to PICOS (participants, interventions, comparisons, outcome and study design).
Methods	5	Protocol and registration	The information of a review protocol could be found, such as web address or its information could be mentioned in the review (e.g., History; Differences between protocol and review), it is considered "yes".
	6	Eligibility criteria	Provided the study characteristics (such as PICOS) used as criteria for eligibility, it is considered "yes".
	7	Information sources	Described all information sources (e.g., databases with dates of coverage, contact with study authors to identify additional studies) in the search and date last searched. It is considered "yes" if all this detailed information has been reported clearly.
	8	Search	If the review did not include any Chinese database, it is considered "yes" when it has reported electronic search strategy used for at least one database. But if any Chinese database was included in the review, it could be at least reported the search strategy of one Chinese database, it is considered "yes".
	9	Study selection	Reported the process for selecting studies (e.g., "Two reviewers independent selecting studies based on eligibility criteria).
	10	Data collection process	Described method of data extraction from reports (such as piloted forms, independently, in duplicate) and any processes for obtaining and confirming data from investigators.
	11	Data items	Listed all variables for which data were sought (such as PICOS, funding sources) and any assumptions and simplifications made.
	12	Risk of bias in individual studies	Described the assessment tool and methods used for assessing risk of bias or methodology quality of individual studies, and how this information is to be used in any data synthesis.
	13	Summary measures	Stated the principal summary measures (such as risk ratio, difference in means).
	14	Synthesis of results	Described the methods of handling data and combining results of studies, if done, including measures of consistency (such as I^2 statistic) for each meta-analysis.
	15	Risk of bias across studies	Reported any assessment of risk of bias that may affect the cumulative evidence (such as publication bias, selective reporting within studies).
	16	Additional analyses	Described methods of additional analyses (such as sensitivity or subgroup analyses, meta-regression), if done, indicating which were pre-specified

Results	17	Study selection	Provided the numbers of studies screened, and included in the review, with reasons for exclusions after reading full text. For a flow diagram, it is considered "yes".
	18	Study characteristics	For each study, presented characteristics for which data were extracted (such as study size, PICOS, follow-up period) and provide the citations. If tables of characteristics of included studies were provided in the review, it is considered "yes". In addition, if there was no included primary study, it is considered "yes" as the tables of characteristics of excluded were provided.
	19	Risk of bias within studies	Provided data on risk of bias of each study. The table or risk of bias summary should be considered "yes".
	20	Results of individual studies	Provided the result related to every outcome. For a forest plot, it is considered "yes".
	21	Synthesis of results	Presented results of each meta-analysis done, including confidence intervals and measures of consistency.
	22	Risk of bias across studies	Provided the information about publication bias or selective reporting within studies. Although publication bias wasn't performed, its reason could be found, it is considered "yes".
	23	Additional analysis	Give results of additional analyses, if done (such as sensitivity or subgroup analyses, meta-regression). It is considered "yes" if the review had described the reason that did not conduct sensitivity or subgroup analyses.
Discussion	24	Summary of evidence	Summarized the main findings including the strength of evidence for each main outcome; consider their relevance to key groups.
	25	Limitations	Discussed limitations at study and outcome level (e.g., risk of bias), and at review-level (e.g., incomplete retrieval of identified research, reporting bias). For example, the description of limitations could be found in the section of "Potential biases in the review process" in the CDSRs.
	26	Conclusions	Provided a general interpretation of the results in the context of other evidence, and implications for future research. For example, it is can be found in the section of "Authors' conclusions" in the CDSRs.
Funding	27	Funding	Described sources of funding for the review and other support (such as supply of data). It is better to report the role of funders for the systematic review. This item could be found in the section of "Source of support" in the CDSRs.

Table 2. Assessment rules for the 9-item of CHM-related information

Category	Item	Specifics	Definition of "yes"
**TITLE**	**1.Title**	Was the specific name of CHM reported?	Report the specific name of CHM in the title, for single herb interventions, such as Oral Astragalus (Huang qi), Danshen (Chinese medicinal herb); for CHM formulas interventions, such as Wendan decoction (Traditional Chinese medicine), Chinese herbal medicine suxiao jiuxin wan. It is considered "yes" (e.g. scored as "1") if the title of an SR had reported a specific name of CHM.
		Others (e.g. generalized name of CHM)	However, in some CDSRs, their titles had been reported as the generalized names of CHM interventions. For example, the names of CHM were "Chinese herbal medicines", "herbal medicines", "herbal preparations", "medicinal herbs", "traditional Chinese Medicine herbs". These are impossible to identify what specific types of the CHM were included in an SR.
		Others (e.g. multiple interventions including CHM)	In addition, few of included SRs had reported their titles as "Interventions", "Complementary therapies", which represented multiple interventions. When you review the full-text of these SR, it is calculated into this category if there included CHM interventions in the SR.
**INTRODUCTIO N**	**2.Rationale**	Was the CHM-related rationale reported from the perspective of TCM theory?	In the CDSRs, the section of "Background" included the introduction and rationale of the "condition", "intervention", and "how the intervention might work", etc. It is considered as "yes" if this part of an SR had reported TCM theory related to the condition and/or intervention, no matter more or less.
**METHODS**	**3.Information source**	Was the search strategy reported Chinese database(s) and/or Chinese medical journals?	It is considered as "yes" if an SR had reported at least one Chinese database and/or had hand-searched Chinese medical journals and/or CHM-related publications of any Chinese pharmaceutical company. For details, we have calculated the numbers in different categories of information source, including database, journals and companies. In addition, we also calculated the number of SRs that had not searched any Chinese database or journals.
	**4.Eligibility criteria for participants**	Was the TCM diagnostic criteria (e.g. pattern/syndrome criteria) of participants reported?	The TCM diagnostic criteria includes TCM disease(s) and/or pattern(s). It is considered as "yes" if an SR had reported any names of TCM diagnostic criteria of syndrome/pattern in the section of "Type of participants" of "Criteria for considering studies for this review" of the "Methods" of an CDSR.
	**5.Eligibility criteria for outcomes**	Was the TCM-related outcome (e.g. tongue, pulse and symptoms) reported?	In the section of "Type of outcome measures" of "Criteria for considering studies for this review" of the "Methods" of an CDSR, it is considered as "yes" if the TCM-related outcome(s) had been reported, either in primary or secondary outcomes. The TCM-related outcomes

			included changes in TCM signs and symptoms, such as participant's tongue and pulses, TCM syndrome scores, etc.
**RESULTS**	**6.Study characteristics**	For the CHM interventions, was the following details reported: composition and dosage, type, dosage form, source, administration route, time of administration, and quality control of the CHM. For the comparisons, was the description of the control groups adequately reported?	First, the eight subitems of the CHM interventions had been calculated respectively. For composition and dosage, it is considered as "yes" if each composition with its dosage had been reported; it is given as "0" if the author had only reported the compositions without specific dosage (except some patent that had reported brand details). For the type of CHM, it is considered as "yes" if an SR had reported clearly the CHM interventions extracted from each primary clinical trial either as single herbs or formulas. For the dosage form, it is considered as "yes" if an SR had reported clearly the dosage form of the CHM intervention(s) extracted from each primary clinical trial. For the source of CHM, it is considered as "yes" if an SR: 1) for CHM formula(s), it had reported the source (e.g. originated from the Ancient, Experience, Agreement or other source of literature, secret recipe, etc.); or 2) for single herb(s), it had reported the source of some of the herbs, if applicable. For the route and time of administration, it is considered as "yes" if an SR had reported clearly this information of each included primary trial. For the quality control of CHM, it is considered as "yes" if an SR had reported this information of some of the included primary trials, no matter more or less. For the control groups, it is considered as "yes" if an SR had reported the name, dose, duration, administration of the basic information of the comparisons. In this study, there are 97 CDSRs that included primary RCTs. So, we calculated the percentage of this item was based on the total number of 97.
	**7. Additional analyses**	Were the considerations of CHM characteristics in the subgroup analysis, sensitivity analysis or other analysis of clinical heterogeneity related to CHM interventions reported?	Characteristics of CHM-related information include CHM types, dosages, forms, sources, etc., which summarized in the above item. These factors should be considered in the additional analysis (e.g. subgroup analysis, or other analysis of clinical heterogeneity). For example, it is considered as "yes" if an SR had reported subgroup analyses under the different types of CHM formulas (standard formulation and individual formulation); or an SR had investigated the sources of heterogeneity from the specifics of the CHM intervention, including the dosage, formulation and quality control of CHM preparations (e.g. herb source, purity; preparation facilities).
	**8.Synthesis of results**	Whether the data synthesis, especially about the meta-analyses had been properly conducted or not?	Although all included SRs reported the statistical methods of handling data and combining results, this study re-assessed whether the synthesis of results in each SR was properly conducted. This assessment was done according to the reporting quality of the above items,

			especially about 6) and 7). Of 109 included SRs, 85 had conducted meta-analyses. Thus, the percentage of this item was based on the total number of 85. The criteria of “properly conducted” was according to the homogeneity of the PICO (e.g. participant, intervention, comparison and outcome) information, especially the reporting quality of the details of CHM interventions and additional analyses provided as above. For example, if some of the CHM-related information was not reported (e.g. CHM composition, dosage, source or quality control information), it is impossible to assess whether the meta-analyses in the SRs were properly conducted or not. Specifically, it is considered as “yes” if an SR had completely reported the above 6) and 7) item specifics. If an SR had not reported enough information to ensure that no heterogeneity was existed in the CHM interventions, but it had conducted the meta-analyses. This is considered as “unclear” that represents “impossible to assess”.
**DISCUSSION**	**9.Summary of evidence and limitations**	Was the discussion reporting the interpretation from the perspective of TCM theory?	For CDSRs, the “Discussion” includes some sections of “Summary of main results”, “Overall completeness and applicability of evidence”, “Quality of the evidence”, and “Potential biases in the review process”, etc. Anywhere had reported discussion contents related to TCM theories has been considered as “yes”. No matter more or less of its words.
